# Glyphosate, Hard Water and Nephrotoxic Metals: Are They the Culprits Behind the Epidemic of Chronic Kidney Disease of Unknown Etiology in Sri Lanka?

**DOI:** 10.3390/ijerph110202125

**Published:** 2014-02-20

**Authors:** Channa Jayasumana, Sarath Gunatilake, Priyantha Senanayake

**Affiliations:** 1 Department of Pharmacology, Faculty of Medicine, Rajarata University, Anuradhapura 50008, Sri Lanka; 2 Health Science Department, California State University, Long Beach, CA 90840, USA; E-Mail: sarathg@csulb.edu; 3 Hela Suwaya Organization, Malabe 10115, Sri Lanka; E-Mail: helasuwaya@gmail.com

**Keywords:** chronic kidney disease of unknown etiology, glyphosate, hard water, nephrotoxic metals, arsenic

## Abstract

The current chronic kidney disease epidemic, the major health issue in the rice paddy farming areas in Sri Lanka has been the subject of many scientific and political debates over the last decade. Although there is no agreement among scientists about the etiology of the disease, a majority of them has concluded that this is a toxic nephropathy. None of the hypotheses put forward so far could explain coherently the totality of clinical, biochemical, histopathological findings, and the unique geographical distribution of the disease and its appearance in the mid-1990s. A strong association between the consumption of hard water and the occurrence of this special kidney disease has been observed, but the relationship has not been explained consistently. Here, we have hypothesized the association of using glyphosate, the most widely used herbicide in the disease endemic area and its unique metal chelating properties. The possible role played by glyphosate-metal complexes in this epidemic has not been given any serious consideration by investigators for the last two decades. Furthermore, it may explain similar kidney disease epidemics observed in Andra Pradesh (India) and Central America. Although glyphosate alone does not cause an epidemic of chronic kidney disease, it seems to have acquired the ability to destroy the renal tissues of thousands of farmers when it forms complexes with a localized geo environmental factor (hardness) and nephrotoxic metals.

## 1. Introduction

### 1.1. Chronic Kidney Disease of Unknown Etiology (CKDu) in Sri Lanka

Starting in the mid 1990s, a Chronic Kidney Disease of Unknown etiology (CKDu) was discovered among the rice paddy farmers in the North Central Province (NCP) of Sri Lanka [1]. Over the next two decades, the disease spread rapidly to the other farming areas. The age-standardized prevalence of the disease is estimated at 15% [2] affecting a total population of 400,000 patients with an estimated death toll of around 20,000 [3]. The unique feature of this CKDu is that its etiology does not include commonly known risk factors for CKD such as diabetes mellitus, hypertension and glomerular nephritis [4]. In 2009, the Sri Lankan Ministry of Health introduced criteria for case definition of CKDu [5]. These included:

(1) No past history of, or current treatment for diabetes mellitus or chronic and/or severe hypertension, snake bites, urological disease of known etiology or glomerulonephritis.
(2) Normal glycosylated hemoglobin levels (HbA1C ˂ 6.5%).
(3) Blood pressure ˂160/100 mmHg untreated or ˂140/90 mmHg on up to two antihypertensive agents.

The CKDu is a disease that progresses slowly [1]. Patients are asymptomatic during most of the course of the disease. Histopathological findings have shown tubular interstitial nephritis associated with mononuclear cell infiltration, glomerular sclerosis and tubular atrophy [6]. The disease is characterized by tubular proteinurea, usually alpha-1 and beta-2 microglobulinuria, and high urine Neutrophil Gelatinase-associated lipocalin (NGal) levels (>300 ng/mg creatinine) [7,8]. The observed geographical and socioeconomic disease patterns led to assumptions that environmental and occupational factors have an important role to play as the main causative agents [9,10]. Tubulointerstitial disease with negative immunofluorescence for IgG, IgM and complement-3 are more in favor of a toxic nephropathy [4], but commonly known nephrotoxins such as lead (Pb), non-steroidal anti-inflammatory drugs, aminoglycosides, aristolochic acid and mycotoxins are highly unlikely as a single cause of CKDu in Sri Lanka. Many victims of CKDu are not aware of being ill until the end stage and their only treatment options are peritoneal and hemodialysis and ultimately, kidney transplantation.

A number of research groups, including the World Health Organization (WHO), have conducted research studies to determine the etiology of this unique type of CKD. There is some consensus that this is a multifactorial disease. The main factors include chronic exposure to arsenic (As) [1], cadmium (Cd) [11] and pesticides [2,12]. Consumption of hard water, low water intake and exposure to high temperatures resulting in significant dehydration, are among the other factors. Whatever hypothesis that is propounded should be able to answer the questions as to why CKDu is confined to certain geographical areas of Sri Lanka and why there was no CKDu in Sri Lanka prior to the 1990s. 

### 1.2. CKDu and Ground Water Hardness

**Figure 1:**
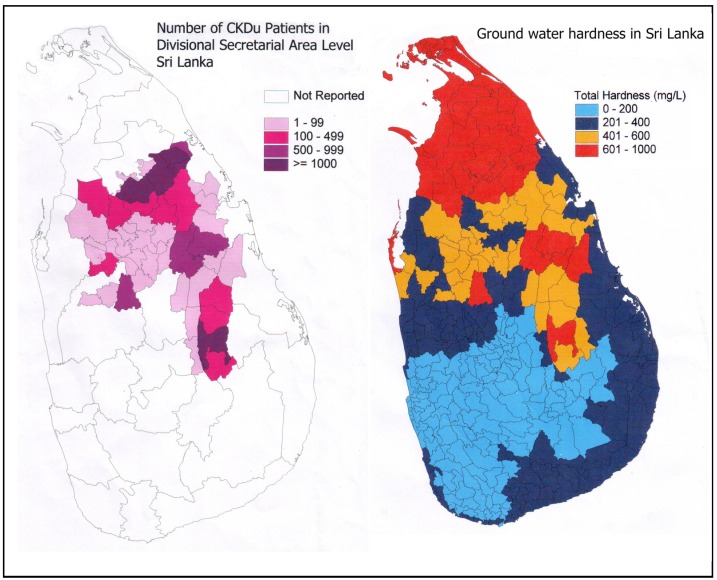
Geographical distribution of patients with CKDu and ground water hardness in Sri Lanka. Ground water hardness data- with the courtesy of Water Resources Board of Sri Lanka.

Places with high ground water hardness and the geographical distribution of the CKDu in Sri Lanka are well correlated (Figure 1). Hardness of water is caused mainly due to the presence of the polyvalent metallic cations calcium (Ca), magnesium (Mg), strontium (Sr) and iron (Fe), together with carbonate, bicarbonate, sulphate and chloride anions [13]. The degree of hardness is classified as, soft, moderately hard, hard or very hard when the Ca and Mg content is 0–60 mg/L, 61–120 mg/L, 121–180 mg/L and >181 mg/L, respectively [14]. Ground water in the CKDu endemic area is found to be either hard or very hard and contain Ca, Mg, Fe and Sr ions [15].

A highly statistically significant positive correlation (*p* < 0.008) has already been revealed between the occurrence of CKDu in Sri Lanka and hard water consumption. Ninety six percent of the CKDu patients had consumed hard or very hard water for at least five years, from wells that receive their supply from shallow regolith aquifers [16]. Apart from that, the authors have made the following observations related to CKDu and the hardness of the drinking water:

(a) The number of villagers who complain that the ground water hardness in CKDu endemic area has increased steadily over the last two decades.
(b) Certain shallow wells (2–5 m), which were previously been used for drinking purposes are now abandoned due to high hardness and bad taste.
(c) There are a few natural springs located in the CKDu endemic area where water is not hard. People who consume water from these sources have been determined to be free from the disease.
(d) Individuals who drink treated water from large water supply schemes (especially in the two cities of Anuradhapura and Polonnaruwa), while living in the same endemic areas, do not have the disease.
(e) In the adjoining farming areas of the Northern Province of Sri Lanka, where the ground water hardness level is known to also be hard or very hard, there have not been any significant number of CKDu cases reported.

Many scientists who have been involved in research related to CKDu have neglected the hard water factor, as there is no scientific evidence linking CKD to the consumption of hard water, or the presence of high Ca or high Mg levels in drinking water. Nevertheless, due to the strong association between hard water consumption and CKDu, certain researchers have attempted to link hard water with a number of other factors related to CKDu. Jayasumana *et al*. [1] have demonstrated that there is a link between hardness and arsenic toxicity. They have identified toxic levels of arsenic in urine, hair and nail samples of CKDu patients as well as in apparently healthy individuals living in the CKDu endemic region. They proposed that arsenic, derived mainly from tainted agrochemicals (chemical fertilizers and pesticides), when combined with calcium and/or magnesium in the ground water can ultimately damage the kidney tissues. Even though there is considerable evidence to suggest that the agricultural workers in the CKDu endemic areas are exposed to arsenic, the exact source and mode of entry of arsenic remains controversial. However, the totality of scientific evidence gathered so far has highlighted the fact that an unknown factor (Compound X) originating from agrochemicals, when combined with hardness/Ca/Mg can cause significant kidney damage; thus explaining many current observations including the unique geographical distribution of the disease. 

## 2. Compound X

If we assume that the “Compound X” is derived from the agrochemicals and is easily bound to Ca/Mg/Sr/Fe to ultimately cause damage to the kidneys, then this hypothesis can explain the geographical distribution of CKDu as well as the occurrence of the disease only after the 1990s. Political changes instituted in 1977 in Sri Lanka, have lead to economic policies that allowed the importation and application of agrochemicals on a large scale, especially for paddy farming. The low concentration of a cumulative nephrotoxin and its bioaccumulation could have taken 12–15 years to cause damage to the kidneys leading up to the level of clinically identifiable CKD. The increase in prevalence of CKDu and the shifting of age at diagnosis to younger age groups over the years are highly suggestive of the cumulative nature of the toxin. Furthermore, a comparatively low amount of agrochemicals has been used in the Northern Province of Sri Lanka, primarily due to a prohibition imposed by the government in this province. The prohibition was due to the potential of these agrochemicals being used in the production of Improvised Explosive Devices (IEDs). These IEDs were used abundantly by armed groups of the terrorist movement that plagued the country until 2009 for causing mass destruction. This is the explanation for the fact that CKDu is still not prevalent in the farming areas of the Northern Province of Sri Lanka where the ground water hardness has remained high. Based upon these observations, here we summarizes the expected properties of the chemical Compound “X” that is hypothesized as the incriminating agent of CKDu.

(a) A compound made of recently (2–3 decades) introduced chemicals to the CKDu endemic area.
(b) Ability to form stable complexes with hard water.
(c) Ability to capture and retain arsenic and nephrotoxic metals and act as a “carrier” in delivering these toxins to the kidney.
(d) Possible multiple routes of exposure: ingestion, dermal and respiratory absorption.
(e) Not having a significant first pass effect when complexed with hard water.
(f) Presenting difficulties in identification when using conventional analytical methods.

The present authors have continuously searched for a possibility of Compound X over the time period of interest and noticed that aminophosphonic acid or aminophosphonate (known by the common chemical name glyphosate) is the most widely used herbicide in the contemporary world [17] as well as in Sri Lanka. The amount of glyphosate exceeded the sum of all other pesticides imported into Sri Lanka in 2012 (Table 1) [18]. The former Stauffer Chemical Company (Westport, CT, USA) initially obtained a patent for aminophosphonic acid as a chelating agent, wetting agent and biologically active compound [19]. Glyphosate was initially used as a descaling agent to clean out calcium and other mineral deposits in pipes and boilers of residential and commercial hot water systems. Descaling agents are effective metal binders, which grab on to Ca, Mg, *etc.* ions and make the metal water soluble and easily removable. Later, the Monsanto Company has acquired the chemical from Stauffer and obtained a patent for aminophosphonate for its herbicidal properties [20].

**Table 1:** Leading Pesticides imported to Sri Lanka in 2012.

Rank	Pesticide	kg or L Approved for Import
01	Glyphosate (acid equivalent)	5,295,082
02	Propanil	995,310
03	MCPA	686,375
04	Mancozeb	671,504
05	Chlorpyrifos	420,008
06	Carbofuran	299,000
07	Diazinon	196,735
08	Profenofos	140,768
09	Carbosulfan	107,000
10	Pretilachlor + Pyribenzoxim	102,297

### 2.1. Glyphosate

**Figure 2:**
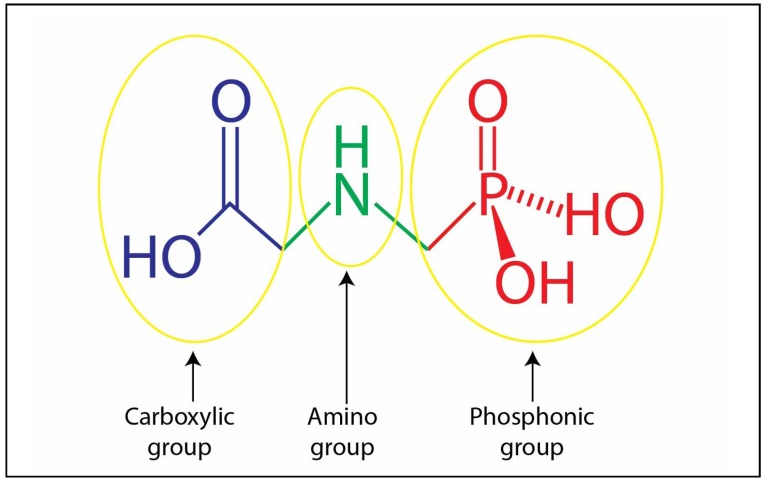
Structure of glyphosate molecule and its functional groups.

Glyphosate or N-(phosphonomethyl)glycine is the aminophosphonic acid analog of the natural amino acid glycine. It was supposed to be first synthesized by Henri Martin in 1950 [21]. The name glyphosate is derived from the word [Gly]cine [phos]phon[ate]. The Monsanto Company acquired another patent for the phytotoxicant properties of N-(phosphonomethyl) glycine [22]. Glyphosate was quickly adopted by almost all farming communities throughout the World and was hailed as the magical total weed killer. In fact, glyphosate was acclaimed as the pesticide of the turn of the millennium and as the most significant chemical in modern agriculture [21]. Glyphosate is a compound with an amphoteric and zwitterion structure containing a basic secondary amino function in the middle of the molecule, monobasic-carboxylic and dibasic phosphonic acidic sites at both ends, hence having three functional groups, phosphonate, amino and carboxylic [23] (Figure 2). A zwitterion is a neutral molecule with positive and negative electrical charges at different locations within the same molecule. It is different from simple amphoteric compounds that might only form either a cationic or anionic species depending on external conditions—a zwitterion simultaneously has both ionic states within the same molecule [24].

Further, glyphosate contains both hydrogen cation donor and acceptor functional groups and has excellent water solubility 12,000 mg/L [25]. The generally accepted mechanism of action of glyphosate is that it inhibits the enzyme 5-enolpyruvyl-shikimate-3-phosphate synthase (EPSPS) of the shikimate pathway in the biosynthesis of tryptophan, phenylalanine and tyrosine (aromatic amino acids) [26]. This pathway is present in plants, fungi and bacteria, but not in animals. Apart from the excellent water solubility and basipetal translocation ability (capability of transportation in the plant from the leaves towards the stem) [21] glyphosate is considered as the best herbicide ever discovered as it is readily degraded to non-toxic degradation products [27]. However, these claims have been debated and Monsanto Company was fined in a legal case with New York Attorney General’s office in 1996 as it had inaccurately represented the toxicological data of the glyphosate in its formulated product “Roundup”. In this case the Monsanto Company agreed to leave out the description of being “environmentally friendly and biodegradable” from its advertisements [28].

**Figure 3:**
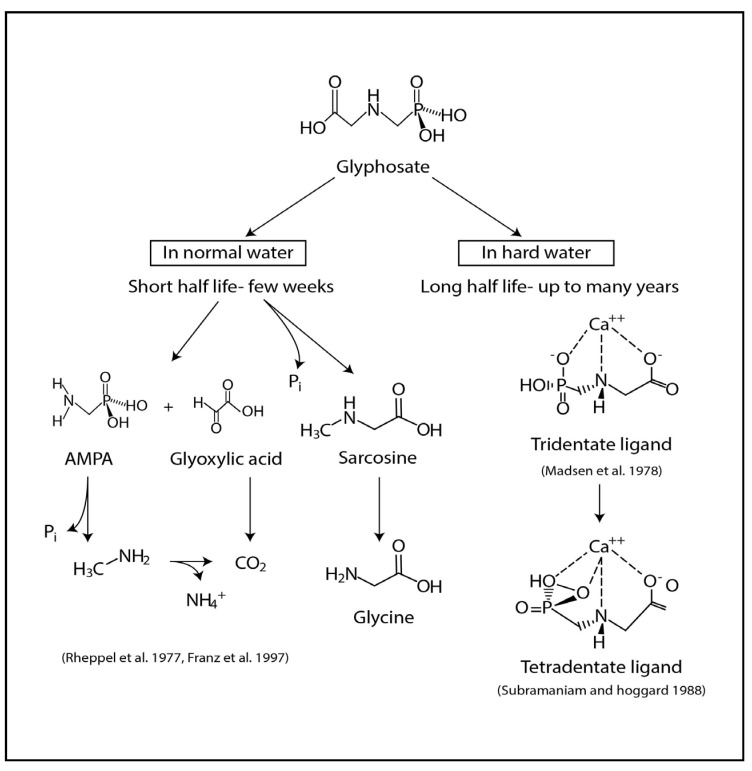
Degradation pathways of glyphosate in normal water and in hard water.

The stability of glyphosate in water or soil depends on several factors. It interacts strongly with soil components by forming stable complexes with metal ions. Adsorption is strongly influenced by cations associated with the soil [29] as it is mainly the phosphonic acid moiety that participates in this process. Therefore, phosphate, which is a component of most fertilizers, competes with glyphosate in soil adsorption [30]. The typical half life of the glyphosate was found to be 92 days in water and 47 days in soil [31,32]. However, the absorption of chelating agents or metals has been shown to decrease the biodegradability of glyphosate (Figure 3) [23,27,33,34,35]. Radioactive ^14^C-glyphosate studies have shown that half-life can increase up to 7 years [36] or even up to 22 years [37] in the soil. Glyphosate is a dianion in moderately buffered soils and water systems when the pH is higher than 6.5. This suggests that under such conditions glyphosate will form strong complexes with metal ions [35]. The increased solubility of its alkali metal glyphosate can leach into deep soil layers [38]. Further, it has been shown that amino methyl phosphonic acid (AMPA) the primary metabolite of glyphosate is more mobile in the soil than the parent compound [39,40]. Detection of glyphosate in the laboratory is very difficult due to its ionic character, high polarity, high solubility in water, low volatility, insolubility in organic solvents and strong complexion behavior [41]. 

### 2.2. Glyphosate-metal Complex (GMC)

**Figure 4:**
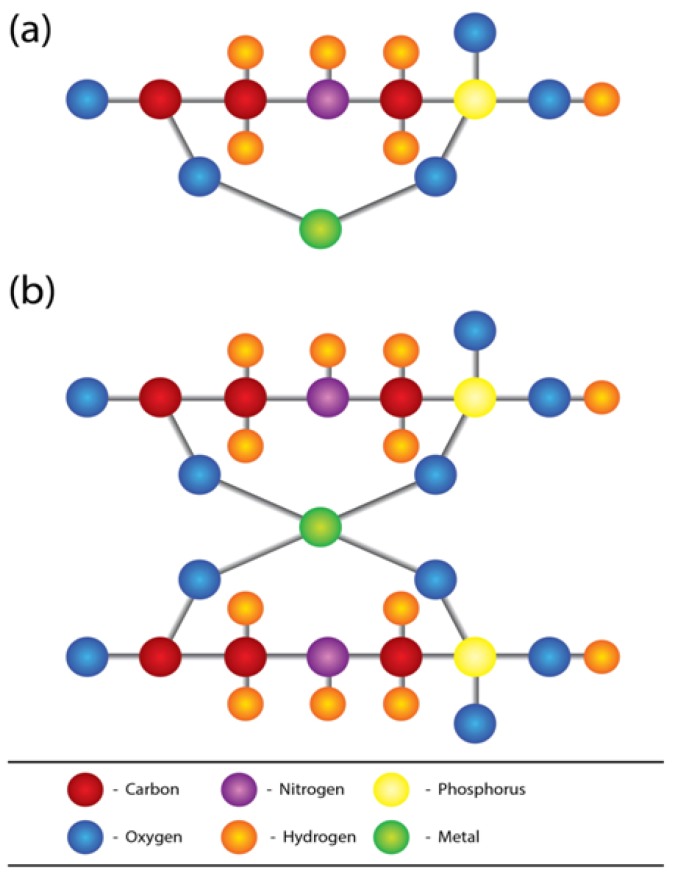
Structures of complexes formed by (**a**) one molecule (**b**) two molecules of glyphosate and metal.

Glyphosate-hard water/Ca/Mg interaction has been the subject of many scientific studies. The negative influence of hard water on the herbicidal properties of glyphosate is a well-identified problem in terms of the efficacy of its weed control [35,42,43,44,45,46,47]. Several measures have also been identified to overcome the antagonism of spray carrier water hardness of glyphosate [48,49]. These strategies mainly depend on the stability of GMC in different pH values. Usually this GMC is stable in basic media and unstable in acidic media. Smith and Raymond 1987 [50] have studied the solid state and solution chemistry of calcium glyphosate. They have isolated the polymeric chemical structure of the compound by using single crystal X-ray diffraction. All the adsorption, photodegradation and biodegradation processes of glyphosate are modified by the presence of metal ions [51]. Nuclear magnetic resonance (NMR) studies done by Thelan *et al* indicate the hard water cations Ca and Mg interact with both phosphonate and carboxyl functional groups of the glyphosate molecule [46]. Further, they have shown that over time, the association of the cations with glyphosate progress to a more structured chelate stable orientation. Glyphosate not only forms stable complexes with Ca and Mg, but also with many other divalent and trivalent metallic cations (Figure 4). Caetano *et al*. [52] assessed the stability of glyphosate—metal complexes and found that the strength of the stability of divalent cations is in the order, Zn > Cu > Ca > Mg and for trivalent cations, Co > Fe > Cr > Al, respectively. In the same study, the authors extensively studied the stability of tetrahedral and octahedral glyphosate-metal complexes as well.

When we go back to the CKDu situation in Sri Lanka and hypothesize that glyphosate is “Compound X”, we can explain almost all of the above-mentioned observations coherently. It provides rational answers for the geographical distribution of the CKDu and the appearance of the disease in the mid-1990s. Glyphosate and its primary metabolite AMPA can directly leach into the ground water and easily chelate to Ca, Mg and Sr copiously present in ground water in the North Central Province and adjacent rice paddy farming areas in the Sri Lanka. Many farmers use hard water to dissolve glyphosate to prepare the spraying solutions as well. Further it is reported that rice paddy farming soil in CKDu endemic area is rich with Ca, Mg, Fe, Cr, Nickel (Ni), Co and other metals [53,54]. It can easily combine with glyphosate and form complexes, which later leach into the ground water. Ferric ions also play a significant role in the process of adsorption of glyphosate and AMPA in soil [55]. Furthermore, within a couple of weeks after the spraying of glyphosate farmers apply triple phosphate (TSP) to the paddy fields. Recent findings have shown that the TSP available in Sri Lanka is contaminated with significant amounts of Cd, Cr, Ni and Pb [54]. Divalent cations of these nephrotoxic metals are capable of forming stable compounds with glyphosate [35]. Furthermore, it was also found that TSP used in Sri Lanka is a very rich source of arsenic [56].

Other modes of ingestion of glyphosate are dermal and respiratory. Low levels of glyphosate have frequently been detected in the urine of farm workers shortly after the glyphosate application [57]. Farmers in Sri Lanka spray pesticides manually under hot climatic conditions. Glyphosate preparations are easily dissolved in sweat and absorbed transdermally [58]. As the majority of farmers do not use any protective gear, absorption through the respiratory route may also play a significant role. Rice is the staple diet of farmers. Recent findings have revealed that rice, vegetables and raw tobacco available in the CKDu endemic areas are contaminated with Cd and As [2]. Chewing of betel with tobacco is a common practice among farmers in Sri Lanka. The phosphorous atom in the phosphonic group in the glyphosate/AMPA molecule can possibly be replaced by As [59,60]. Following dermal and respiratory absorption of glyphosate, it can form complexes with nephrotoxic metals and As derived from rice, vegetables and tobacco within the circulation. As such, we can identify three potential sources of glyphosate/AMPA-metal complexes:

(a) [Glyphosate/AMPA + Ca/Mg/Fe/Sr ] complex in drinking water.
(b) [Glyphosate/AMPA + Cd/Cr/Ni/Co/Pb/Vanadium (V) or As] complex in food.
(c) [Glyphosate/AMPA coming from dermal/ respiratory route] + low amount of [metals/As] from water and foods, here the complex is formed within circulation.

**Figure 5:**
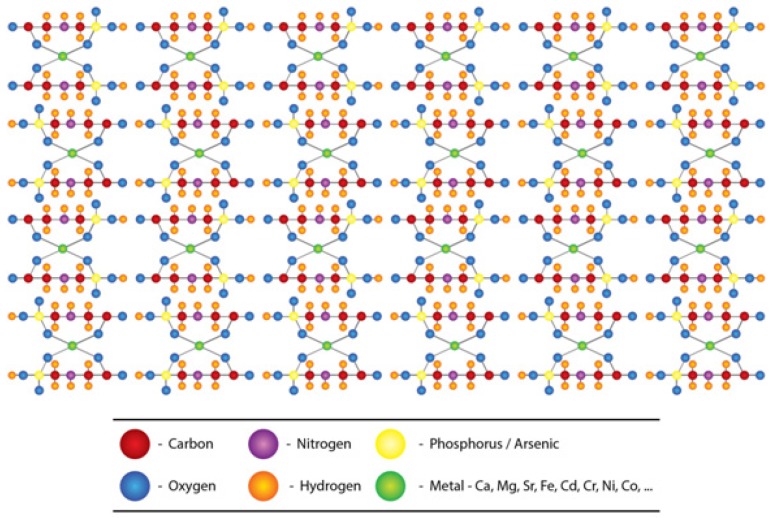
Structure of glyphosate-metal-arsenic lattice.

Helfter Enterprises, Inc. now doing business as Advanced Biological Concepts has proposed a structure for glyphosate matrix [61], while Caetano and coworkers [52] have developed a more advanced and comprehensive structure for glyphosate metal complexes. The latter group has used density functional theory (DFT) molecular modeling methods to evaluate structural thermodynamic and electronic effects that govern the complexion between glyphosate and metals. With the permission of both groups of authors, we used these structures to propose a glyphosate-metal lattice to explain the possible role played by glyphosate, hardness, As and other nephrotoxic metals in the pathogenicity of CKDu in Sri Lanka (Figure 5).

This hypothesis also explains the observation of increased ground water hardness in paddy farming areas in Sri Lanka. Various divalent and trivalent metal glyphosate compounds accumulate in ground water over the years and made ground water more hard and distasteful. Natural springs located in the CKDu endemic area are devoid of high Ca and Mg content, hence these natural springs do not retain glyphosate. In light of this explanation, it is reasonable to hypothesize that glyphosate-metal complex plays a major role in the CKDu disease process. It explains why CKDu is not present among people who drink natural spring water or surface water in the disease endemic area. Also the limited use of herbicide and chemical fertilizers in the northern region over the last few decades may be the reason for lack of CKDu there despite the consumption of hard water by the inhabitants in this area.

### 2.3. The Nephrotoxicity of Glyphosate-metal/As (GMA) Lattice

The next important question to be answered is whether the glyphosate-metal complex is nephrotoxic or not. Nephrotoxicity of As, Cd and other heavy metals is a known fact [62]. Many studies have been conducted to assess the activity of Ca-glyphosate, Mg-glyphosate behavior in soil water and in plants [38]. The majority of them have been targeted to overcome the antagonistic ability of Ca/Mg on glyphosate [63]. Although glyphosate has a history of more than 40 years of usage as an herbicide and it has been almost 50 years since the identification of hardness-aminophosphonic acid reaction, none of the available studies has focused on the animal or human health effects of hardness-glyphosate complex. However, glyphosate alone has been the subject of several animal studies.

Jiraunghoorskul *et al.* [64] described changes in proximal tubular cells of Nile Tilapia exposed to glyphosate. Ayoola [65] has shown the development of hematopoietic necrosis and severe pyknotic nuclei, dilatation of bowman’s space, accumulation hyaline droplets in tubular epithelial cells in the proximal tubule and degenerated tubules in juvenile African catfish exposed to glyphosate. Seralini and others [66] have shown in a long term study that glyphosate increased serum creatinine, blood urea and reduced the weight of kidneys of rats who were fed with glyphosate exposed maize. Tizhe *et al.* [67] have provided further confirmation that oral exposure of glyphosate increases blood urea levels and lead to renal dysfunction in rats. Larsen *et al.* [68] have described the glutathione peroxidase dependent reduction of cumenehydroperoxide in kidneys of rats exposed to glyphosate in drinking water. Kruger *et al.* [69] has shown a similar nephrotoxic effect in dairy cows exposed to glyphosate. Although EPSPS and the shikimate pathways are not present in animals, the inhibition of other pathways such as cytochrome p450 and aromatase is the possible explanation of genotoxic [70] and teratogenic [71] activity of glyphosate and the dose dependent effects of round up on human embryonic and placental cells [72]. Glyphosate has also been documented to induce apoptosis and necrosis in human umbilical, embryonic and placental cells [73] and cause endocrine disruptive effects on human cell lines [74]. 

### 2.4. Compound X-elusiveness of Detection by Standard Tests

Persistence of glyphosate in water have previously been reported [75,76]. In a recent study done in Catalonia, Spanish researchers reported that glyphosate was present above the limits of detection in 41% of the ground water samples obtained from areas where intense agricultural activities had taken place [77]. Another Spanish study has shown that certain chelating agents when present in ground water can produce false negative results for glyphosate tests, however, the same phenomenon was not observed in the case of surface water [78]. These researchers found that acidification of ground water samples to a level of pH 1 can lead to significant changes in the final readings of the glyphosate tests. Difficulty in the analysis of glyphosate and AMPA in the presence of multivalent cations was demonstrated in a study done in France [79]. In this study, investigators have shown that only the free forms of glyphosate and AMPA are sensitive to analytical methods and exact concentration is underestimated particularly in ground water. In Europe 0.1 μg/L is administratively set as the upper tolerable level for all the pesticides, including glyphosate in drinking water [80].

### 2.5. Lack of a Significant First Pass Effect

Once the glyphosate-metal-As lattice enters the circulation it may bypass the normal liver detoxification process. Usually divalent metal transporter-1 (DMT-1) mediates absorption of heavy metals in the small intestine [81]. Thereafter, it is transported to the liver and binds with metallothioneins (MTs)—a protein with high content of cystine [82]. The main function of MTs is to transfer heavy metals to various metalloproteins, transcription factors, and enzymes [83]. Here, we hypothesize that the liver cannot metabolize the GMA lattice due to its unique configuration. The structure of cystine closely resembles that of glycine [84]. Glyphosate/AMPA is the aminophosphonic analog of the natural amino acid glycine [21]. As the heavy metals are already bound to glyphosate/AMPA the binding sites that would have normally attracted MTs are already occupied. As such, these GMA complexes pass through the liver without a significant first pass effect. This assumption also explains the normal liver enzyme levels and minimal ultrasonic changes in the liver of patients with CKDu. Once GMA lattice reaches the kidney, the glomerular-proximal tubular area provides a distinctive microenvironment conducive to the breakdown of the lattice. Differences in the pH and the presence of various metabolic products provide this background. Kidneys excrete 50–100 meq/day of non-carbonic acid generated daily. This is achieved by H^+^ ion secretion at different levels in the nephron. The entire daily acid load cannot be excreted as free H^+^ ions. Secreted H^+^ ions are excreted by binding to either buffers, such as HPO_4_^2−^ and creatinine, or to NH_3_ to form ammonium ions (NH_4_^+^). Ammonium is produced from glutamine in the proximal tubule [85,86,87]. NH^+^_4_ ions have been used for many decades by agricultural experts to minimize the binding of glyphosate to hard water which effectively decreases the availability of the active weedicide [88]. 

Further, in analytical chemistry acidification is used as an effective dissociation method of glyphosate/AMPA complexes to obtain free forms [78]. Therefore, we have further hypothesized that this high concentration of the NH^+^_4_ ions that releases the heavy metal from the GMA lattice in the proximal tubular area.

**Figure 6:**
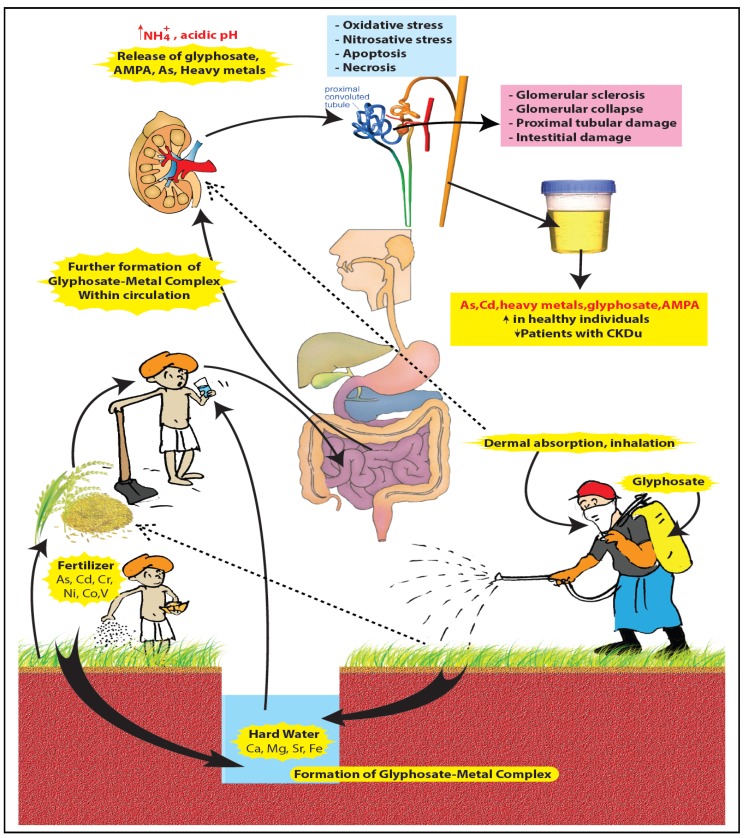
GMA lattice hypothesis in summary.

When lattice is broken down, it releases metals and arsenic. Excessive amount of glyphosate/AMPA and As may start the damage to the glomeruli while As, Cd, Cr, Ni, Co, Pb, V are reabsorbed up to a certain extent at the proximal tubules resulting in further tubular damage. Long-term exposure to these substances causes oxidative stress, nitrosative stress, apoptosis and necrosis [89,90,91] in the glomerular and proximal tubular cells. Glomerular sclerosis, glomerular collapse and tubular interstitial damage are the result of these pathological mechanisms (Figure 6). Several animal studies have already demonstrated the reduction of GFR in chronic toxin (adriamycin) induced nephropathy associated with the development of both tubulointerstitial nephritis and glomerular sclerosis [92]. Furthermore, Javaid and coworkers [93] have shown that the reduction of GFR is closely correlated with the extent to which glomeruli are no longer connected to the normal tubules. They suggest that a local extension of glomerular injury to destroy the tubular neck is an important cause of loss of renal functions. If we apply the same model to CKDu this explains the comparatively low level of urinary excretion of creatinine, As, Cd, Cr, Ni, Co, V and glyphosate by CKDu patients (Cases) when compared to healthy individuals in the same family or living in the same endemic area (Controls) (unpublished data produced at the California State University, Long Beach, CA, USA). Presence of high levels of As and Cd in nail and hair samples of CKDu cases as compared to the controls [1,2] is confirmatory evidence of the exposure and accumulation of As and Cd in the body as the kidneys become increasingly incapable of excreting them. Destruction of the tubular necks following long term exposure to GMA lattice also brings about a sudden decrease of renal functions in the later stages of the CKDu which result in the death of the patient if dialysis or renal transplantation is not done.

## 3. CKDu Elsewhere

A CKDu epidemic very similar to that of Sri Lanka has been identified among the paddy farmers in Andra Pradesh—a southeastern province of India [94]. These authors reported that ground water is the only available water source in Uddanam and Chikamurthy, two of the areas with the highest CKDu prevalence. Analysis of samples of drinking water revealed that metal ions and trace elements in drinking water were within allowable limits, and thus not expected to lead to any deleterious effects on human health. However, in these findings it was clearly shown that the total hardness, Ca, Mg and Sr values are quite high. Especially in Chikamurthy area, some of the drinking water samples exceed 1,000 mg/L of total hardness. The authors may not have paid enough attention to this finding as hardness is not identified as a nephrotoxin or as causing significant human health problems, apart from being a suggested risk factor for exacerbation of eczema [13]. This is exactly the same situation that happened in Sri Lanka. The Sri Lanka Ministry of Health and the WHO conducted a joint investigation and an evaluation of CKDu in Sri Lanka from 2008 to 2013. In the third progress report of the WHO handed over to the Ministry of Health Sri Lanka on 19 February 2012, it has been mentioned that the waters in the 99% of the sources used by patients with CKDu are hard to very hard [95]. However, this factor has not received any further attention when the WHO and the Ministry of Health produced their final scientific publication [2]. The inability to detect glyphosate-metal complexes using the commonly used analytical methods may have deterred the investigators in both Sri Lanka and Andra Pradesh from looking further into the role of these compounds in CKDu.

An epidemic of tubular nephropathy has been identified among young male farm workers in sub-regions of the Pacific coasts of the Central American (CA) countries of El Salvador, Nicaragua and Costa Rica [96,97]. Like the Sri Lankan and Indian scenarios, the etiology is not linked to the most frequent causes of CKD such as diabetes mellitus and hypertension. Rubio *et al.* [98] estimated a death toll of at least 20,000 in the CA region for the last two decades. In El Salvador, hospitalization for CKD increased by 50 percent from 2005 to 2012 and today, it has become the leading cause of hospital deaths. A total of 39,000 of hospitalized cases of CKDu in El Salvador were reported, while 1,474 of them were below the age of 20 years [99]. Clinical, biochemical and histopathological characteristics of CKDu in both Sri Lanka and CA shares a very similar pattern [100]. Therefore, it’s logical to argue that the etiologies in both regions could have many commonalities. The disease is common in sugarcane cultivating areas in CA where some of them previously used to grow cotton [101]. Both sugarcane and rice belong to the grass family and need a comparatively higher amount of agrochemicals in large-scale cultivation [102]. Glyphosate is the leading pesticide used in El Salvador as well [103]. If we apply the same hypothesis to explain the CKDu in CA it can logically explain the occurrence of disease among male farm workers in pacific coastal line. The CA Pacific coastal line belongs to the volcanic belt [104,105]. In this region soil and groundwater naturally contain high amounts of metals and As [106]. These levels of As could be additive to the As which originated from fertilizers and agrochemicals as pesticides with inorganic As were commonly used in cotton cultivation. When sugarcane became the leading crop in the Pacific coastal line after 1990s [107], this crop could have used huge amounts of glyphosate, 2,4-D and other pesticides. These conditions make it highly the suitable for the formation of a GMA lattice in ground water and soil with the consequent bioaccumulation in people living in this area. The El Salvador National Institute of Health also confirmed that the water from shallow wells had been the main drinking water source for the majority of CKDu patients in the country. Furthermore, they have detected significant amounts of hardness, As and heavy metal levels in their water samples [108].

## 4. Glyphosate as “Compound-X”—Available Evidence and Areas for Further Research

To prove that glyphosate is the “Compound-X” that chelates with calcium and the other metals to become the causative agent of CKDu, one has to establish a clear chain of evidence. The first link in this chain is a well-established fact as shown earlier–that is, glyphosate is a strong metal chelator (for Ca, Mg, Sr, Cd, Cr, Ni, Co, Pb); It is immobilized in soil by chelating with soil cations; It persists and accumulates in soil and plants for extended periods (years)–therefore, these immobilized chelates can contaminate the water table.

The second link in the hypothesis is to confirm that the water from the wells which the CKDu patients have used is contaminated with glyphosate and metal ions. In another study that is ongoing at California State University, Long Beach, CA, USA we have tested water samples (n = 50) from these contaminated wells and found that almost all of them contained glyphosate with high content of Ca and other metals. The authors had to use a special Enzyme Linked Immuno-Sorbent Assay (ELISA) test to detect these glyphosate-metal complexes, as they are not amenable to the conventional analytical methods. Glyphosate and heavy metals were also found in the urine of both CKDu patients as well as the control subjects who lived in the CKDu endemic area. This is not surprising as most of the controls drank water from the same wells. Therefore, we have confirmatory proof on the ingestion of these complexes in drinking water and excretion of components of the complex in urine. The manner in which the glyphosate metal complexes are absorbed through the intestines needs further research, perhaps beginning with animal models. None of the CKDu patients (n = 125) showed any significant elevation of liver enzymes or ultrasound evidence of detectable liver pathology. This is the best evidence that we have so far about the escape from the first pass metabolism by the glyphosate metal complexes. This is the same reason why we see more renal manifestations in As poisoning of CKDu patients. However, we occasionally see the classic cutaneous and liver manifestations only in some CKDu patients with advanced renal damage [1]. Acquavella *et al.* [57] have demonstrated how the glyphosate excretion increased in 48 farmers and their families after spraying. However, they have not separately assessed the contributions of the dermal and respiratory routes of exposure. Further research should be undertaken to study how glyphosate is absorbed into the circulation through dermal and respiratory routes, particularly after spraying the pesticide.

The third link in this hypothesis is how the glyphosate metal complex contributes to renal damage. From current renal physiology it is well known that ammonium ions are generated in proximal tubule. In fact, this is the principle component of the acid excretion of the kidney [109]. It is also well known from USA studies that ammonium sulphate is used as a buffer to release glyphosate bound to metal ions [88]. Therefore, it is plausible to assume that this same mechanism is in effect in the proximal tubule. However further research including renal biopsies and animal studies are necessary to confirm that this is actually the same mechanism that is at work within the renal tubules.

## 5. Conclusions

CKDu, the major health issue in the rice paddy farming areas in Sri Lanka, has been the subject of many scientific and political debates over the last decade. Although there is no agreement among scientists about the etiology of the disease, a majority of them have concluded that this is a toxic nephropathy. None of the hypotheses put forward so far could explain coherently the totality of clinical, biochemical, histopathological findings, and the unique geographical distribution of the disease and its appearance since the mid 1990s.

The strong association of the consumption of hard water and occurrence of CKDu has been subjected to many discussions among investigators, but none of the available theories could explain this relationship coherently. Here we have explained the association by using glyphosate, the most widely used herbicide in the disease endemic area. The strong metal chelating property of glyphosate and related compounds is a well-known fact. However, the human health effects of glyphosate-metal complexes have not been given any serious consideration by investigators for last four decades. Huge advertising campaigns by glyphosate as the best ever herbicide discovered by mankind, reiteration of the easily degradable nature of the original compound in a natural environment and the difficulties in the laboratory detection may have been the reasons for this delay. Results being produced through the current study that is ongoing in the California State University, Long Beach are highly supportive of this hypothesis. Stability of glyphosate metal complexes in various environmental conditions and nephrotoxic properties of the compound should be the subjects of further investigation.

The GMA lattice hypothesis gives rational and consistent explanations to the many observations and unanswered questions associated with the mysterious kidney disease in rural Sri Lanka. Furthermore, it may explain the similar epidemics of CKDu observed in Andra Pradesh, India and Central America. Although glyphosate alone does not cause an epidemic of chronic kidney disease, it seems to have acquired the ability to destroy the renal tissues of thousands of farmers when it forms complexes with a localized geo environmental factor (hardness) and nephrotoxic metals. It is logical to find out other agricultural areas in the World where excessive use of glyphosate and drinking ground water with high hardness and the contamination of ground water and food with nephrotoxic metals have overlapped in causing kidney damage.
